# Salivary Cytokines and Airways Disease Severity in Patients with Cystic Fibrosis

**DOI:** 10.3390/diagnostics10040222

**Published:** 2020-04-15

**Authors:** Alice Castaldo, Paola Iacotucci, Vincenzo Carnovale, Roberta Cimino, Renato Liguori, Marika Comegna, Valeria Raia, Gaetano Corso, Giuseppe Castaldo, Monica Gelzo

**Affiliations:** 1 Dipartimento di Scienze Mediche Traslazionali, University of Naples Federico II, 80131 Naples, Italy; ali.castaldo@hotmail.com (A.C.); paola.iacotucci@unina.it (P.I.); vincenzo.carnovale@unina.it (V.C.); valeria.raia@unina.it (V.R.); 2 Dipartimento di Neuroscienze, Scienze Riproduttive ed Odontostomatologiche, University of Naples Federico II, 80131 Naples, Italy; roberta.cimino@unina.it; 3 Dipartimento di Scienze e Tecnologie, University of Naples Parthenope, 80133 Naples, Italy; renato.liguori@uniparthenope.it; 4 Dipartimento di Medicina Molecolare e Biotecnologie Mediche, University of Naples Federico II, 80138 Naples, Italy; marika.comegna@unina.it (M.C.); monica.gelzo@unina.it (M.G.); 5 CEINGE-Biotecnologie Avanzate, 80145 Naples, Italy; 6 Dipartimento di Medicina Clinica e Sperimentale, University of Foggia, 71122 Foggia, Italy; gaetano.corso@unifg.it

**Keywords:** cystic fibrosis, inferior turbinates hypertrophy, nasal polyposis, salivary cytokines

## Abstract

About 50% of patients with cystic fibrosis (CF) have sinonasal complications, which include inferior turbinate hypertrophy (NTH) and/or nasal polyposis (NP), and different degrees of lung disease, which represents the main cause of mortality. Monitoring of sinonasal disease requires complex instrumental procedures, while monitoring of lung inflammation requires invasive collection of bronchoalveolar lavage fluid. The aim of this study was to investigate the associations between salivary cytokines levels and CF-related airway diseases. Salivary biochemical parameters and cytokines, i.e., interleukin-6 (IL-6), IL-8, and tumor necrosis factor alpha (TNF-α), were analyzed in resting saliva from healthy subjects and patients with CF. Patients with CF showed significantly higher levels of salivary chloride, IL-6, IL-8, and TNF-α and lower calcium levels than healthy subjects. Among patients with CF, IL-6 and IL-8 were significantly higher in patients with NTH, while TNF-α was significantly lower in patients with NP. A decreasing trend of TNF-α in patients with severe lung disease was also observed. On the other hand, we did not find significant correlation between cytokine levels and *Pseudomonas aeruginosa* or *Stenotrophomonas maltophilia* colonization. These preliminary results suggest that salivary IL-6 and IL-8 levels increase during the acute phase of sinonasal disease (i.e., NTH), while the end stages of pulmonary disease and sinonasal disease (i.e., NP) show decreased TNF-α levels.

## 1. Introduction

Cystic fibrosis (CF) is an autosomal recessive life-limiting disorder due to mutations of the gene encoding the cystic fibrosis transmembrane conductance regulator (CFTR). The mutated protein causes a variable defect in the transport of sodium and chloride through epithelial cells of the respiratory, biliary, gastrointestinal, and reproductive tracts, giving rise to secretions of thick mucus [1]. Both lower and upper airways are typically involved [2,3]. Lung disease is the principal cause of morbidity and mortality in CF [4,5]. From the first months of life, infection and inflammation trigger lung disease [6]. Moreover, patients with CF may have chronic recurrent sinusitis, hypertrophy of the inferior turbinates (NTH), and nasal polyposis (NP) with nasal airway obstruction [7]. Sinonasal symptoms are frequently underestimated in CF patients due to the severity of other clinical issues [8].

Several procedures have been employed to detect respiratory infections and to analyze inflammatory biomarkers in patients with CF, e.g., bronchoalveolar lavage fluid (BALF), nasal swab, cough swab, and induced sputum [9]. Children are not able to expectorate the sputum; thus, clinical monitoring of lung disease in children with CF requires the invasive collection of BALF [10,11].

The analysis of salivary biomarkers could represent a non-invasive alternative approach [12]. Passive collection (resting saliva) is the most recommended method, although sample volume is usually lower than that obtained from stimulated collection [12]. In recent years, some studies have been carried out aiming to identify new biomarkers of CF such as electrolytes [13,14] and cytokines [15] in saliva from patients with CF. However, these studies have been performed on stimulated saliva, while there are no studies on the analysis of biomarkers from resting saliva.

In particular, interleukin-6 (IL-6), IL-8, and TNF-α represent the principal pro-inflammatory cytokines detected in CF airway epithelia [16]. Furthermore, an increase of IL-8 and IL-5 was observed in the nasal secretions of patients with CF compared to healthy subjects, but no significant differences were found between patients with and without NP [17]. In addition, Nunes et al. [18] reported similar mRNA levels of IL-8 and IL-6 in CF patients with and without NP. On the other hand, the cytokine profile in airway fluids from CF patients with NTH and the differences of cytokine profile between CF patients with NP and NTH have not been well investigated.

The principal aim of this study was to evaluate the associations between salivary cytokine levels and CF-related airway diseases. To this end, we measured first the levels of biochemical parameters and cytokines, i.e., IL-6, IL-8, and tumor necrosis factor-alpha (TNF-α) in resting saliva samples from adult patients with CF compared to healthy subjects. Second, the salivary levels of cytokines were related to the following: (1) severity of sinonasal disease, (2) severity of pulmonary disease, and (3) the presence of *P. aeruginosa* (PA) or *S. maltophilia* (SM) colonization.

## 2. Materials and Methods

### 2.1. Study Population

We recruited 129 adults with CF at the Adult Regional Center for Cystic Fibrosis (see Table 1 for demographic data) and 50 healthy volunteers with a median age (interquartile range) of 31 (26–38). The study was approved on 24 December 2015 (code: 244_2015) by the ethics committee of Federico II University Hospital and was performed according to the current version of the Helsinki Declaration. Written informed consent was obtained from all patients. The diagnosis of CF was performed according to current guidelines [19]. The CFTR genotype was defined through the screening of the most frequent mutations and rearrangements [20]. CFTR gene sequencing was performed when mutations were not detected in one or both alleles [21].

### 2.2. Sample Collection

Whole resting saliva samples (obtained without mechanical or chemical stimulation) were collected between 9 a.m. and 12 a.m. The patients were instructed not to drink or eat anything except water for 2 h before the sample collection. One to three milliliters of whole resting saliva were collected in sterile plastic tubes, which were chilled on ice during saliva collection. Immediately after collection, the samples were centrifuged for 30 min at 14,000× *g* to remove bacteria/cellular debris and the supernatants were stored at −80 °C.

### 2.3. Clinical Evaluation

All CF patients underwent nasal endoscopy to evaluate the clinical status of the nasal cavities (i.e., presence or absence of NP and NTH) [22].

Lung disease was classified as severe, moderate, or mild, considering both the age and the most recent forced expiratory volume in 1 s (FEV1) of patients while they were clinically stable [23]. The FEV1 was expressed as the percentage of predicted value (% pred) for age, according to standardized reference equations for spirometry [24]. Airway colonization by PA or other bacteria was identified by sputum or oropharyngeal swab culture. Pancreatic insufficiency was defined on the basis of fecal pancreatic elastase, one fewer than 200 μg/g feces measured in the absence of acute pancreatitis or gastrointestinal diseases.

### 2.4. Salivary Biochemical Parameters

Salivary concentrations of chloride and potassium were measured using Integrated Chip Technology (Abbott Diagnostics, Rome, Italy). All other assays were performed using reagent kits provided by Abbott Diagnostics with an automated biochemistry analyzer (Architect ci16200 Integrated System, Abbott Diagnostics, Rome, Italy). In particular, salivary calcium and phosphate were measured by colorimetric methods. Salivary lactate dehydrogenase (LDH) was analyzed by the specific enzymatic assay. Total proteins were measured by the urine/CSF protein method.

### 2.5. Enzyme-Linked Immunosorbent Assay (ELISA)

Salivary cytokines were analyzed using human IL-6, IL-8, and TNF-α ELISA Max™ Set Deluxe kits (BioLegend, Inc., San Diego, CA, USA). One day prior to carrying out the assay, 96-well plates were coated with the capture antibody and incubated overnight at 4 °C. The plates were then washed four times with PBS containing 0.05% Tween-20 (Sigma-Aldrich, St. Louis, MO, USA) and incubated for 1 h at room temperature with diluent A as the blocking buffer. After four washings, 100 μL of saliva (or diluted standard solutions for calibration curve) was added to each well and then incubated for 2 h at room temperature with shaking. After four washings, 100 μL of biotinylated detection antibody solution was added, and the samples were incubated for 1 h. After another four washings, 100 μL of avidin-horseradish peroxidase solution was added and incubated for 30 min. Following five washings, 100 μL of 3,3′,5,5′-tetramethylbenzidine substrate solution was added, and the samples were incubated for 15 min in the dark. The reaction was stopped by the addition of 100 μL of 2N sulfuric acid, and the absorbance at 450 nm and 570 nm was measured. The absorbance at 570 nm was subtracted from that at 450 nm. All samples and standard solutions were analyzed in duplicate and the mean absorbance was calculated. The concentration values (pg/mL) of each cytokine were obtained by interpolating the absorbance values on the respective calibration curve. The average of CV%, calculated from all standard and sample duplicates, was less than 4.5% (SD: 3.5%).

### 2.6. Statistical Analysis

Continuous data were reported as mean and standard deviation (SD) for parametric distributions or median and interquartile range for non-parametric distributions. The Shapiro–Wilk test was used to verify the normality of distributions. Categorical data were reported as frequency and percentage. Comparisons between two groups were performed by t-test or by non-parametric Mann–Whitney U test as appropriate. Statistical differences between three groups were assessed by ANOVA or a non-parametric Kruskal–Wallis test. Post-hoc pairwise, multiple comparisons were performed using Bonferroni’s correction or a non-parametric Mann–Whitney U test as appropriate. Significance was accepted at the level of *p* < 0.05. Box plots were created by KaleidaGraph software (version 4.1.1, Synergy, Reading, PA, USA).

## 3. Results

Table 1 describes demographic and clinical data of 129 adults with CF.

Salivary biochemical analysis revealed that chloride concentrations were significantly higher in CF patients than in controls, while calcium and phosphate concentrations were lower (Table 2).

As shown in Table 3, all the three salivary cytokines, i.e., IL-6, IL-8, and TNF-α, were significantly higher in CF patients than in the controls.

**Figure 1:**
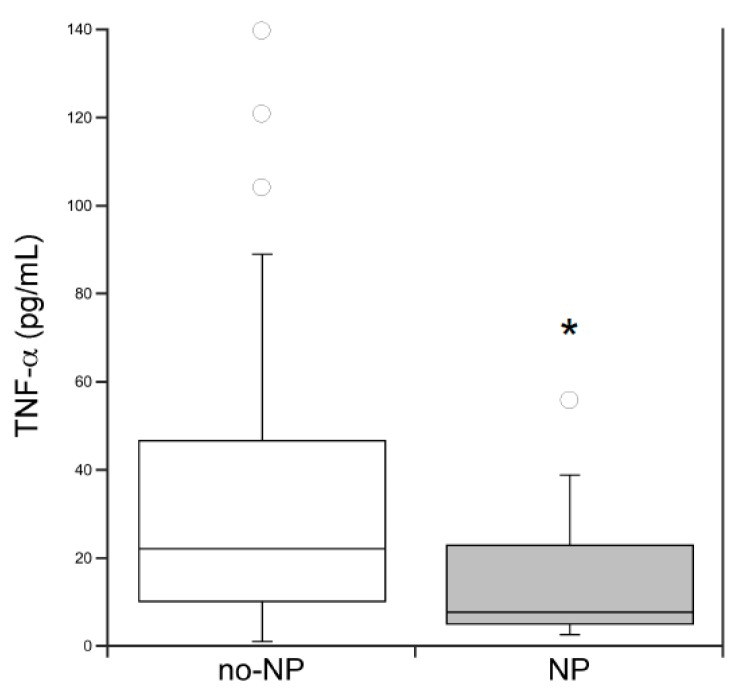
Comparison of salivary TNF-α levels between CF patients without nasal polyposis (no-NP: no-NTH/no-NP + NTH; *n* = 108) and with nasal polyposis (NP, *n* = 21); * *p* < 0.05, Mann–Whitney U test.

In addition, we compared the salivary cytokines levels among CF patients with NTH and NP and those without NTH/NP (Table 4). The comparisons between the three groups showed that salivary IL-6 and IL-8 levels were significantly higher in patients with NTH. On the other hand, the comparison of TNF-α between patients with NP and those without NP (also including the patients with NTH) showed that salivary TNF-α levels were significantly lower in patients with NP (Figure 1).

Additionally, we compared the salivary cytokines levels among the three sub-groups of patients on the basis of the severity of lung disease, i.e., mild, moderate, or severe (Table 5). We did not observe significant differences between the three sub-groups, although TNF-α levels were lower (with a borderline statistical significance) in the sub-group of patients with severe lung disease compared to moderate.

Finally, there was not a significant difference in the comparison of salivary cytokines between CF patients with and without PA or SM colonization (Table 6).

## 4. Discussion

The levels of some biochemical parameters as well as the levels of IL-6, IL-8, and TNF-α in saliva are significantly different between patients with CF and the healthy controls; furthermore, salivary levels of cytokines correlate with the severity of pulmonary and sinonasal disease. However, the diagnostic use of saliva as a biological matrix requires the standardization of some pre-analytical variables, such as collection methods, i.e., resting or stimulated saliva, and circadian variations [12]. Before starting the study, we compared salivary electrolytes between resting and stimulated saliva samples, and we did not observe statistical differences (data not shown) in agreement with a previous study [25]. In all subjects we collected resting saliva samples in the morning (between 9 and 12 a.m.).

We observed higher chloride concentrations in saliva from patients with CF compared to healthy subjects (in agreement with previous data obtained on stimulated saliva), which depends on the altered CFTR function [13,14]. Moreover, we observed that salivary calcium is significantly lower in CF patients compared to healthy subjects. This finding is in agreement with a previous study on stimulated saliva in which Gonçalves et al. [13] found slightly lower levels of calcium in CF patients. The reduction of salivary calcium observed in patients with CF could be due to the enhanced calcium influx in CF airway cells [26]. The authors showed that the abnormal calcium influx depends on the transient receptor potential canonical (TRPC)-6, which is functionally and reciprocally joined to CFTR in epithelial human airway cells. In CF cells, this complex is altered, causing elevated intracellular calcium levels, which in turn leads to IL-8 secretion in CF airways [27]. In fact, we observed that saliva of patients with CF contains significantly higher levels of IL-8.

We found a significant increase of IL-6, IL-8, and TNF-α in saliva from CF patients as compared to the controls. Such cytokines are the principal pro-inflammatory modulators detected in CF airway epithelia [16], and various studies have suggested a dysregulated production of IL-8 in CF respiratory epithelial cells even without bacteria colonization [28,29]. Lung inflammation often precedes infection in the course of CF lung disease, and several studies found increased levels of pro-inflammatory cytokines in the sputum and BALF from patients with CF, although often without correlations to the respective blood levels and clinical status [16]. In fact, we did not observe correlations between salivary cytokines levels and lung colonization.

Among the patients with CF, we found significantly higher levels of IL-6 and IL-8 in patients with NTH. Accordingly, Kenney et al. [30] detected IL-6 and IL-8 mRNA in whole tissue turbinates and isolated epithelium, as well as in supernatants of epithelial cell cultures. Therefore, in sinonasal acute inflammation, as in NTH [31], there could be an enhanced production and/or secretion of IL-6 and IL-8. At the early stage of inflammation, a positive feedback of inflammatory signals also led to the production of anti-inflammatory cytokines, and it seems that in CF, the balance between pro- and anti-inflammatory signals is altered, causing IL-8 overproduction [29].

In this study, no differences of salivary IL-8 and IL-6 were observed between CF patients with NP and without NTH/NP. Accordingly, a cross-sectional study reported similar levels of IL-8 and IL-6 mRNA in CF patients with and without NP [18]. We observed that salivary TNF-α was significantly reduced in patients with NP, which is the most advanced phase of the sinonasal disease. In addition, we found a decreasing trend of TNF-α in patients with severe lung disease. TNF-α is a potent pro-inflammatory cytokine and is involved in the primary steps of acute inflammation. Subsequently, in an inflammatory state that lasts over time, TNF-α stimulates IL-6 synthesis that, besides pro-inflammatory actions, exerts a negative feedback on acute inflammatory response by inhibiting TNF-α production [32]. In chronic inflammation, as in NP, macrophages contribute to the inflammatory process by chronically producing low levels of TNF-α, causing some clinical symptoms, such as anorexia and cachexia, which are also observed in patients with CF [33].

A limitation of this study is represented by the small number of CF patients with NP as well as CF patients with severe lung disease. Therefore, the low levels of salivary TNF-α observed in these sub-groups of patients should be considered preliminary, and further studies should be performed on a larger number of CF patients to confirm these results. Moreover, we were not able to perform a priori the power analysis for salivary cytokines analyzed in this study, as there was no pilot study. However, a retrospective analysis of the comparison of salivary TNF-α between patients with and without NP showed a power of 80.23%.

## 5. Conclusions

Our preliminary study suggests that resting saliva could represent a valid non-invasive matrix for the investigation of CF-related airway disease. The novelty of this study is that salivary cytokine profiles differ between CF patients with NTH and NP. Specifically, our results suggest that salivary IL-6 and IL-8 levels increase during the acute phase of sinonasal disease (i.e., NTH), while the end stages of pulmonary disease and sinonasal disease (i.e., NP) show decreased TNF-α levels. Further studies are needed to confirm whether these results extend to children patients.

## Figures and Tables

**Table 1:** Demographic and clinical parameters of adult patients with cystic fibrosis (CF).

	CF Patients (*n* = 129)
Age (years) ^a^	28 (23–36)
Gender, males (%)	72 (56.3)
Pancreatic insufficiency, n (%)	71 (55.1)
Lung disease severity, n (%):	
severe	15 (11.6)
moderate	30 (23.3)
mild	84 (65.1)
Colonization, n (%):	
with PA or SM	71 (55.0)
with PA alone	65 (50.4)
with SM alone	2 (1.6)
no PA or SM	58 (50.0)
Nasal polyposis, n (%)	21 (16.3)
Nasal turbinate hypertrophy, n (%)	31 (24.0)

^a^ Median (interquartile range); PA: *P. aeruginosa*; SM: *S. maltophilia*.

**Table 2:** Salivary biochemical parameters in control subjects and in adult patients with CF.

Analytes	Controls (*n* = 50)	*p* Value	CF Patients (*n* = 129)
K^+^ (mmol/L)	20.4 (8.9–32.8)	n.s.	18.0 (9.5–27.4)
Ca^2+^ (mg/dL)	5.2 (2.0–7.7)	2.3 × 10^−6^	2.8 (2.0–6.1)
Cl^−^ (mmol/L)	20 (20–31)	0.02	22 (20–55)
Phosphate (mg/dL) ^a^	14.7 (6.1)	0.01	11.1 (3.2)
LDH (UL)	128 (30–639)	n.s.	57 (30–689)
Protein (mg/dL)	51.2 (24.3–123)	n.s.	50.6 (17.3–194)

Data with non-parametric distributions are reported as the median (interquartile range), and the comparisons were performed by Mann–Whitney U test; ^a^ Data with normal distribution are reported as mean (SD), and the comparison was performed by t-test; LDH: lactate dehydrogenase. ns: not significant difference.

**Table 3:** Salivary IL-6, IL-8, and TNF-α levels in control subjects and in adult patients with CF.

Cytokines (pg/mL) ^a^	Controls (*n* = 50)	*p* Value	CF Patients (*n* = 129)
IL-6	28.2 (14.0–47.2)	4.3 × 10^−8^	49.5 (40.4–70.1)
IL-8	55.0 (28.8–81.3)	3.2 × 10^−15^	300 (130–514)
TNF-α	12.4 (7.6–26.4)	0.042	19.9 (7.6–39.0)

^a^ Data are reported as the median (interquartile range), and the comparisons were performed by Mann Whitney U test.

**Table 4:** Salivary IL-6, IL-8, and TNF-α levels in CF patients with and without inferior turbinate hypertrophy (NTH) and nasal polyposis (NP).

Cytokines (pg/mL)	no-NTH/no-NP (*n* = 77)	NTH (*n* = 31)	NP (*n* = 21)	*p* Value
IL-6	55.3 (36.0–75.7)	67.0 (45.5–86.1) ^a^	43.5 (30.8–49.5)	0.036
IL-8	388 (261)	566 (386) ^b^	387 (282)	0.028
TNF-α	20.0 (9.4–47.0)	16.6 (5.5–30.8)	7.9 (5.2–23.1)	n.s.

Data with non-parametric distributions are reported as the median (interquartile range), and data with normal distribution are reported as mean (SD); ^a^ *p* = 0.005, NTH versus NP; ^b^ *p* = 0.023, NTH versus no-NTH/no-NP. n.s.: not significant difference.

**Table 5:** Salivary IL-6, IL-8, and TNF-α levels in CF patients with mild, moderate, and severe lung disease.

Cytokines (pg/mL)	Mild (*n* = 84)	Moderate (*n* = 30)	Severe (*n* = 15)	*p* Value
IL-6	54.4 (38.4–81.1)	52.8 (36.7–62.2)	61.4 (45.6–68.0)	n.s.
IL-8	413 (304)	429 (308)	506 (323)	n.s.
TNF-α	19.7 (6.2–38.5)	29.3 (19.6–51.8)	9.9 (6.0–23.0) ^a^	n.s.

Data with non-parametric distributions are reported as the median (interquartile range), and data with normal distribution are reported as mean (SD). ^a^ *p* = 0.050, severe versus moderate. n.s.: not significant difference.

**Table 6:** Salivary IL-6, IL-8, and TNF-α levels in CF patients with *P. aeruginosa* (PA) or *S. maltophilia* (SM) and without PA/SM colonization

Cytokines ^a^ (pg/mL)	no-PA/no-SM (*n* = 58)	*p* Value	PA or SM (*n* = 71)
IL-6	54.4 (40.6–87.6)	n.s.	56.3 (36.6–69.1)
IL-8	323 (176–582)	n.s.	402 (223–580)
TNF-α	21.5 (6.2–38.6)	n.s.	19.7 (7.4–39.0)

^a^ Data are reported as the median (interquartile range), and the comparisons were performed by Mann–Whitney U test. n.s.: not significant difference.
